# Creation of a cardiotropic adeno-associated virus: the story of viral directed evolution

**DOI:** 10.1186/1743-422X-10-50

**Published:** 2013-02-11

**Authors:** Lin Yang, Xiao Xiao

**Affiliations:** 1 Wuhan Institute of Virology, Chinese Academy of Sciences, 44 Xiaohongshan, Wuhan, 430071, Hubei, China; 2 Division of Molecular Pharmaceutics, UNC Eshelman School of Pharmacy, 2070 Genetic Medicine Building CB#7362, Chapel Hill, NC, 27599, USA

**Keywords:** Adeno-associated virus, Cardiac gene transfer, Directed evolution, Transduction

## Abstract

Adeno-associated virus (AAV) is an important vector system for human gene therapy. Although use of AAV serotypes can result in efficient myocardial gene transfer, improvements in the transduction efficiency and specificity are still required. As a method for artificial modification and selection of gene function, directed evolution has been used for diverse applications in genetic engineering of enzymes and proteins. Since 2000, pioneering work has been performed on directed evolution of viral vectors. We further attempted to evolve the AAV using DNA shuffling and *in vivo* biopanning in a mouse model. An AAVM41 mutant was characterized, which was found to have improved transduction efficiency and specificity in myocardium, an attribute unknown for any natural AAV serotypes. This review focuses on the development of AAV vector for cardiac gene transfer, the history of directed evolution of viral vectors, and our creation of a cardiotropic AAV, which might have implications for the future design and application of viral vectors.

## Introduction

**Figure 1:**
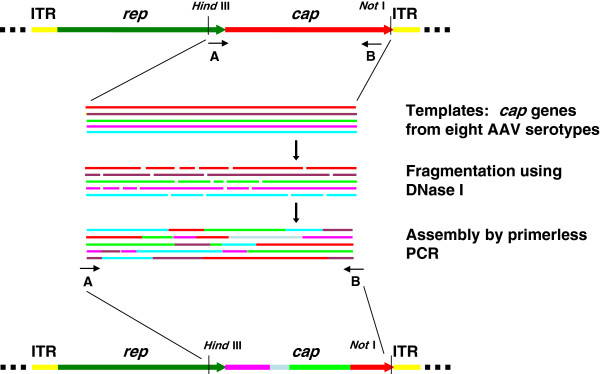
**Modification of adeno-associated virus (AAV) *cap *gene by DNA shuffling.** The *cap* genes of eight AAV serotypes were amplified and mixed together in the same ratio. They were then fragmented by transient DNase I treatment. The small DNA fragments were reassembled by primerless PCR and homologous recombination were introduced in this step. Lastly, the full-length *cap* genes were amplified and inserted into the plasmid vector with AAV2 *rep* gene and *ITR* sequences for generation of plasmid and virus libraries.

The adeno-associated virus (AAV) is one of the smallest viruses, with a non-enveloped capsid measuring approximately 22 nm. It has a linear single-stranded DNA genome of 4.7 kb in size, which is composed of three gene elements: *rep*, which encodes the replication proteins; *cap*, which encodes the capsid proteins; and *ITR*, which serves as the replication origin and packaging signal (Figure 1). The *cap* gene is transcribed from a single p40 promoter, but produces three virion proteins VP1, VP2, and VP3, which differ in length from each other because of the different location of their initiation codons. These three proteins have molecular masses of 87, 72, and 62 kDa, respectively, and they assemble in a molar ratio of 1:1:10 to form a near-spherical protein shell of 60 subunits. Despite the fact that an alternative product of the *cap* gene, associated with the assembly of the AAV particle has been characterized [1], only the three capsid proteins mentioned above are mainly responsible for the tissue tropism and neutralization properties of AAV.

Despite its prevalence rate of approximately 80% in human populations, AAV has not been linked to any human illness, and has thus been used as a vector to carry DNA into cells. AAV transduces cells via a multi-step consecutive course [2,3]. The virus initially binds to the receptor/co-receptor on surface of the cell, and penetrates into it via receptor-mediated endocytosis. The virion is transported through the endosomal compartment, and most likely escapes into the nucleus through the nuclear pore complex via an as yet unknown mechanism. After nuclear translocation, the capsid uncoats, releasing single-stranded DNA, which is converted into double-stranded DNA for transgene expression. The majority of the AAV vector genomes exist as episomes in the transduced cells [4], which ensures the stable expression of the transgene in post-mitotic tissues (e.g., neuron and muscle) with a lower risk of carcinogenesis. Furthermore, the AAV vector itself elicits only a relatively weak innate and acquired immune response in the hosts [5], which affirms the tolerated transgene expression and the higher safety profile in gene therapy. These two biologic features mean that AAV has great potential as a vector for human gene therapy.

The safety and efficacy of AAV-mediated gene therapy were first verified in clinical trials investigating nervous diseases. Sub-retinal delivery of AAV carrying the gene for the retinal pigment epithelium-specific 65 kDa protein (RPE65) was verified by several studies to be safe and effectively improve retinal function in patients with Leber’s congenital amaurosis [6-8]. Gene transfer of glutamic acid decarboxylase via AAV into the subthalamic nucleus of subjects with Parkinson’s disease was well tolerated, and significantly improved the clinical scores of the subjects [9]. More recent clinical trials have expanded the application of the AAV vector to gene therapy of muscular diseases. Calcium upregulation was achieved by intracoronary administration of an AAV1/sarcoplasmic reticulum Ca^2+^-ATPase vector; the study demonstrated safety and suggested a benefit in the patients of advanced heart failure [10]. Long-term, sustainable alpha-sarcoglycan expression of was detected in the muscles of patients with limb-girdle muscular dystrophy following AAV1-mediated gene transfer regulated by the muscle-specific tMCK promoter [11] (more detailed documentation of current progress in clinical trials using AAV vectors can be found in the review by Mingozzi and High [12]).

Despite the numerous advantages of AAV for gene transfer, several flaws remain to be addressed to improve the versatility of this viral vector. In this review, we provide an introduction to the development of AAV vector for myocardial gene transfer, and focus on how a cardiotropic AAV was created using the directed evolution strategy. The related progress of directed evolution is reviewed to provide a background to this discovery.

### Why is a cardiotropic AAV needed? The evolution of AAV vector biology

For historic reasons, the serotype 2 (AAV2) was the first AAV used as a gene-therapy vector and for detailed biological studies [13]. The AAV2 genome was shown to exist in transduced mouse skeletal muscle as concatamers, and the transgene expression persisted for 1.5 years without induction of humoral or cellular immune responses [14,15]. Immediately following this research, AAV2 began to be used for transduction of myocardium in animals. In contrast to the transient transgene expression from adenovirus transduction, up to 50% of mouse cardiomyocytes were found to be positively transduced 4–8 weeks after intramyocardial injection or coronary artery perfusion with AAV2 [16]. Further studies applied the AAV2 vector to therapeutic gene transfer for the treatment of various heart diseases such as myocardial infarction [17,18], dilated cardiomyopathy [19], and dystrophic cardiomyopathy [20]. Generally, these gene-therapy trials yielded favorable results, with stable and efficient transgene expression achieved in the animal myocardium.

In addition to AAV2, a number of AAV serotypes have been successively identified and their attributes characterized for cardiac gene transfer [21]. Of the known serotypes, AAV1 and AAV6 appeared to transduce the rodent myocardium most promptly and efficiently by direct intramyocardial injection [22,23]. Accordingly, AAV6 has been used for delivery of betaARKct, a peptide capable of inhibiting activation of G protein-coupled receptor kinase 2 into rats with heart failure, resulting in a significant improvement of cardiac contractility and reversal of left ventricular remodeling [24]. AAV6 was also first successfully employed to achieve widespread transduction of cardiac and skeletal muscles in adult mice by intravenous administration supplemented with vascular permeability factor [25]. A further technical advancement occurred with the use of AAV8, which appeared to be more capable than AAV6 of crossing the blood-vessel barrier than AAV6, and efficiently transduced heart and skeletal muscles in mice and hamsters without assistance of vascular permeability factor [26,27]. More recently, AAV9 was shown to have an even higher transduction efficiency in mouse myocardium following intravenous injection [28], and a similar result was observed in non-human primates [29]. Notably, the myocardial transduction efficiency of these AAV serotypes were further validated in different heart disease models via systemic delivery [27,30-32]. These studies built a foundation for further application of AAV vectors to translational medicine.

Such studies on various AAV serotypes have greatly improved the performance of the AAV vector in myocardial gene transfer. However, all of the known AAV serotypes tend to transduce the liver preferentially when administered *in vivo*, especially with systemic infusion. This feature is a barrier to safe and efficient cardiac gene transfer in human beings, as transduction of unintended organs might result in unnecessary toxicity and immune response. Fortunately, researchers have begun to address this issue, using a variety of approaches. Müller *et al.* modified the AAV2 capsid by introduction of R484E and R585E mutations to the heparin sulfate proteoglycan binding sites, which improved the specificity of cardiac transduction by AAV vector to some extent [33]. Asokan *et al.* generated a chimera AAV2i8 by replacing the hexapeptide sequence in the heparan sulfate receptor footprint of AAV2 with that of AAV8 [34]. AAV2i8 selectively transduced cardiac and whole-body skeletal muscle tissues with high efficiency, and showed markedly reduced hepatic tropism. However, it still appears challenging to improve the cardiac transduction of AAV vector as well as to detarget it from liver and other unintended organs. We hypothesized that the directed evolution approach might be an option to achieve this aim because of its highly diverse and flexible applications. The related hypothesis and techniques are documented below.

### How can a cardiotropic AAV be created? Hypothesis and techniques for directed evolution of AAV

AAV is a parvovirus that exhibits some unusual evolutionary features. In a series of investigations on evolution of non-human primate AAV, the AAV *cap* genes were found to exhibit unexpectedly high levels of gene diversity [35,36]. Furthermore, this sequence diversity was caused in part by homologous recombination of co-infecting parental viruses that modified the serologic reactivity and tropism of the virus. These studies not only shed light on the evolutionary mechanism for genetic variation in the AAV genome, but also served as the hypothesis for our research on directed evolution of AAV, i.e., that modification of the AAV *cap* genes by homologous recombination could plausibly generate AAV variants with novel tissue tropism. These references also indicated that polymerase chain reaction (PCR) might be a more sensitive molecular technique for retrieval of trace AAV variants from animal tissues in comparison to the classic virus isolation methods based on cell culture.

At the time we began our work, some pioneering studies had been carried out into directed evolution of a retrovirus. Genetic recombination between six murine leukemia viruses was achieved by DNA shuffling, and the resulting virus library was selected using the non-permissive CHOK1 cell line to evolve a completely new tropism [37]. Directed evolution is a method of artificially modifying and selecting genes with desirable properties not found in nature. With respect to DNA shuffling, it was a method originally invented by Stemmer to induce *in vitro* recombination between homologous genes by PCR (Figure 1) [38]. Sequence variants from a gene or orthologous genes are treated with DNase I to generate random DNA fragments. Using PCR amplification with the homologous DNA as templates and primers reciprocally, these fragments are reassembled into genes of the same size as the original, but with variable functions. Homologous recombination between the DNA fragments occurs, and the reassembled genes are eventually cloned for selection on a biological platform for the specific functions required. Although directed evolution had been used previously to engineer proteins or enzymes, the work of Soong and his colleagues on retrovirus was the first attempt to evolve a gene within the framework of a virus, which expanded the biological application of this technique in biomedicine [37].

One classic method to modify the tropism of a viral vector was to insert a ligand on the virus surface to target the specific cell type. Initially, the targeting ligand was isolated via an indirect approach such as peptide phage display, and then inserted into the virus surface for further characterization. Müller *et al*. were the first to attempt direct display of a random peptide library on the AAV surface to select the endothelial-cell-targeting ligands [39]. The peptide library was inserted after the R588 site of the AAV capsid, in order to minimize steric change and assure effective ligand exposition. The authors ensured encoding of each displayed peptide by the packaged DNA by using a two-step system for virus library production. After screening on primary human endothelial cells, the selected AAV with targeting peptides exhibited enhanced transduction of coronary endothelial cells but reduced infection of non-endothelial cells. Although this work did not fall into the classic category of directed evolution, it enriched the concept and practice for diversification and screening of virus library.

The first AAV study using directed evolution in the stricter sense was published by Maheshri *et al.* in 2006 [40]. In that work, the AAV2 *cap* gene was subjected to random mutagenesis, followed by *in vitro* recombination to construct an AAV library with high capsid diversity. The AAV library was eluted from a heparin-binding column, and AAV mutants with different heparin affinity were separated by fractionation. In accordance with its lower heparin affinity, one AAV mutant showed reduced transduction efficiency when heparan sulfate binding became limiting. In the second application, the AAV library was passaged in 293 cells after pre-incubation with anti-AAV neutralizing serum. After two rounds of modification and selection, one AAV clone, r2.15, was found to display a capacity to resist antibody neutralization that was 96-fold higher than that of wild-type AAV2. The modification methods and selection strategies used in this study would exert an important influence on successive directed evolution research on AAV.

### What have we done? The modification and selection of AAV vectors targeting heart and skeletal muscle

The original aim of our work was to modify the AAV vector for efficient detargeting from liver to muscle. The natural evolution of AAV urged us to use artificial evolution to mimic this course in order to improve the transduction efficiency of AAV in striated muscles. As nine AAV serotypes have now been successively identified, we decided to select eight of these (AAV1–4, AAV6–9) for DNA shuffling of the *cap* genes, as AAV5 *cap* genes has been shown to have the largest sequence variation compared with those of other AAV serotypes and might lower the efficiency of *in vitro* recombination. We also expected that using the natural AAV *cap* genes as parents for DNA shuffling would ensure a higher viability of the AAV progenies. The shuffled *cap* genes were cloned into a plasmid backbone with the AAV2 *rep* gene and *ITR* sequences (Figure 1). Random clones were picked from the plasmid library for examination of sequence variation and viability of the recombined AAV *cap* genes. An AAV library was then produced, referring to the two-step method described by Müller *et al*. [39]. The *cap* genotype and phenotype of each virus particle were hyphothetically one-to-one correspondent, which would facilitate the successive screening AAV library.

**Figure 2:**
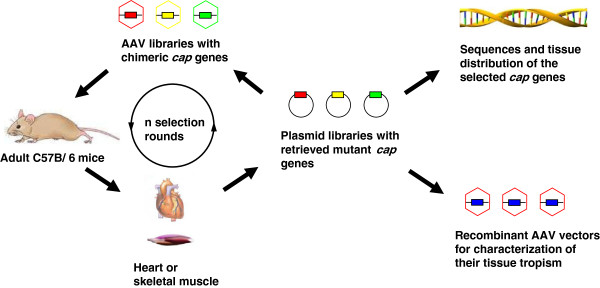
***In vivo *selection of adeno-associated virus (AAV) variants targeting special tissues.** The AAV library with diversified *cap* genes was injected into adult C57BL/6J mice via the tail vein. Genomic DNA was isolated from the target organs, i.e., the heart or skeletal muscle, and used for PCR amplification of AAV *cap* genes. The selected *cap* gene mutants were re-cloned into plasmid vector with AAV *rep* gene and *ITR* sequences. The generated plasmid libraries were used for sequence analysis of *cap* genes and production of AAV libraries for the next screening cycle in mice. After a few rounds of *in vivo* biopanning, the *cap* genes enriched in the heart or skeletal muscle but relatively rare in the liver were used for packaging of recombinant AAV vectors for characterization of their tissue tropism.

We used two models, one *in vitro* and the other *in vivo*, for selection of AAV variants targeting muscles. The *in vitro* model was composed of a microvascular endothelial cell barrier and a differentiated myocyte culture. The *in vivo* model was the C57BL/6J mouse, which as an inbreeding strain has been universally used for biomedical research. We hypothesized that the *in vivo* model would be more realistic, as it should perfectly mimic the physiological environment for the successive gene therapy application of the AAV vector. We first tested the *in vivo* model by using intravenous injection of an AAV library into mice, followed by isolation of the AAV variants from tissues using PCR (Figure 2). We were able to retrieve the AAV *cap* genes from several mouse tissues, including heart, skeletal muscle and liver, for genetic analysis and reconstruction of the virus libraries. Therefore, the C57BL/6J mouse model was appropriate for our purpose to screen AAV variants that could bypass the vascular barrier and infect muscles. When the virus library was injected intravascularly into the mice, we were able to intentionally select those AAV variants preferentially infecting a specific organ, such as the heart.

Our aim was to select AAV variants targeting the heart and skeletal muscle. Accordingly, following the first screening, the AAV *cap* genes were retrieved from both heart and skeletal muscle for reconstruction of the virus libraries for the second screening in mice. The AAV *cap* genes from the heart and skeletal muscle, respectively, were then sequenced, and their gene frequencies were compared with those from the liver. The AAV mutants enriched in heart or skeletal muscle were selected for further genetic analysis and tropism characterization. Strikingly, we obtained a cardiotropic AAVM41 variant from the mouse skeletal muscle [41]. This was not completely unexpected, as cardiac and skeletal muscles share a similar anatomical origin, and are both subordinate to striated muscles. Our variant, AAVM41, exhibited preferential tropism to mouse myocardium after systemic administration, with a transduction level similar to that of AAV9. However, gene transfer in non-muscle tissues, mainly the liver, was dramatically reduced. This higher affinity to heart rather than liver appeared to be a unique feature of AAVM41, and has not been reported for any naturally evolved AAV variant. Furthermore, the therapeutic potential of AAVM41 was demonstrated by delivery of the δ-sarcoglycan gene into the dystrophic TO-2 hamster for efficient amelioration of cardiomyopathy.

**Figure 3:**
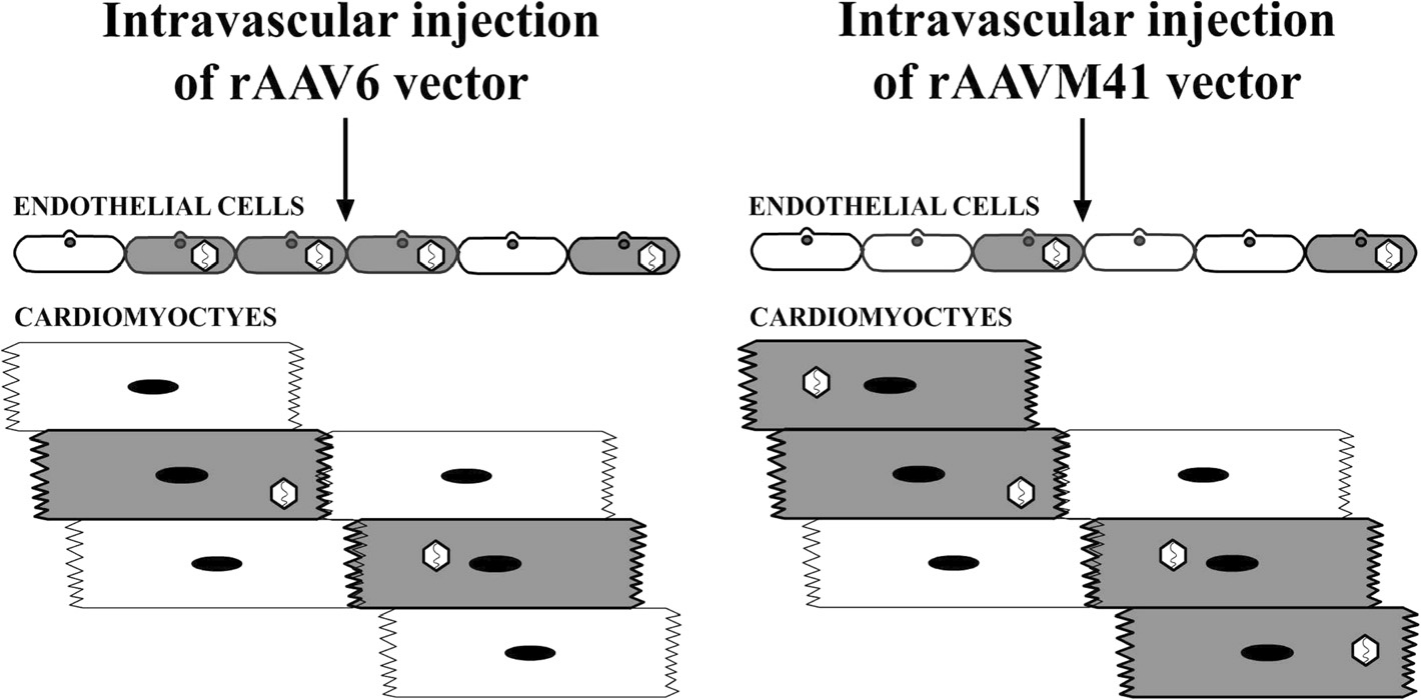
**The putative mechanism for different transduction efficiencies of adeno-associated virus (AAV) types AAV6 and AAVM41 vectors in mouse heart.** These two vectors entered the cardiac tissues with the similar efficiency as a whole. However, AAVM41 might preferentially transduce the cardiomyocytes whereas AAV6 tended to infect endothelial cells with the higher ratio. This discrepancy in tissue distribution enabled AAVM41 to exhibit higher transduction efficiency of myocardium compared with AAV6 after systemic administration. Note that the virus particles in the interior of the cells indicated only that these cells were positively transduced. The real intracellular localization and viral structure of AAV are not reflected in this chart for simplicity.

It remains unclear as to how the AAVM41 vector evolved its improved efficiency and specificity in myocardial transduction. The sequence of the AAVM41 capsid is closest to that of AAV1 and AAV6 [41]. It also had the similar vector genome copies in the whole heart to that of AAV6 after systemic vector administration. However, the myocardial transduction efficiency of AAVM41 was at least 10 times higher than that of AAV6. By contrast, AAV6 was superior to AAVM41 in direct transduction of primary cardiomyocytes *in vitro*. One plausible interpretation is that AAV6 tends to infect both endothelial cells and cardiomyocytes in the *in vivo* physiological environment, whereas AAVM41 could bypass the endothelial cells and preferentially infect the cardiomyocytes when infused into blood stream (Figure 3). Because it is the cardiomyocytes rather than the vascular endothelial cells that occupy the major volume of the heart, this would then account for the fact that AAVM41 generally exhibited a higher transgene expression level in the myocardium. It would be possible to test this hypothesis by comparative study of the vector genome distribution of these two AAV vectors in different cardiac cell types after systemic administration. The recently developed quantitative 3-D tracing microscopic technique will also be helpful to elucidate the mechanism behind the improved cardiac transduction of the AAVM41 vector [42].

### Progress and prospects for directed evolution of AAV

Since the first report of directed evolution of AAV published in 2006, a number of papers have been published in this area. Researchers have designed various strategies for modification of AAV to achieve diverse purposes. Generally, the following development strategies were used.

1. Combinatory application of multiple methods for diversification of the AAV *cap* gene. The original attempts to modify the AAV *cap* gene respectively involved random peptide insertion [39], random mutagenesis [40], and DNA shuffling [41]. In some recent work on directed evolution, AAV libraries with *cap* genes diversified by these methods were simultaneously screened in a suitable cell model. As a result, the ShH19 mutant from combinatory libraries produced by DNA shuffling and random mutagenesis was shown to have the highest transduction efficiency in rat astrocytes *in vivo*[43]. An AAV r3.45 variant with both a peptide insertion and a point mutation was found to have the most efficient transduction of neural stem cells [44]. These results demonstrated the power of simultaneous utilization of multiple gene diversification methods for efficient direct evolution.

2. Realistic biological model for screening of AAV library. In cases where a virus library with high gene diversity has been constructed, the biological platform is likely to be the determining factor for successful directed evolution. An organotypic human airway model was used to screen a virus library produced by the diversification of AAV2 and AAV5 *cap* genes, and it was found that the AAV2.5 T mutant yielded was able to mediate gene transfer more than 100-fold better than parental strains, and corrected the cystic fibrosis epithelial Cl^−^ transport defect [45]. Furthermore, mouse models were used to evolve AAV targeting tumors or internal organs from random peptide display libraries [46,47]. The animal models demonstrated efficacy in improving either the transduction efficiency or specificity of AAV variants, but not both, in these *in vivo* studies. A realistic biological model closely mimicking the environment for gene therapy application would fulfill the purpose for vector modification more successfully.

3. Combination of directed evolution and rational design. Usually directed evolution and rational design were implemented in parallel for modification of viral vectors. However, in one recent piece of research, bioinformatics analysis was used to select a few loops from the AAV capsid for subsequent semi-random sequence mutagenesis [43]. The resulting AAV random loop library was then used for selection of AAV variants targeting human astrocytes. A second research group first introduced random mutagenesis into the GH loop spanning amino acids 390–627 of the AAV9 capsid [48]. Based on structural analysis, variants with deleterious mutations were triaged for further work, and only AAV clones with potentially beneficial mutations were characterized for vectors detargeting away from the liver. These two reports respectively employed bioinformatic clues for construction of the AAV library and structural analysis for directed selection of AAV variants, which may have important implications for directed evolution work in the future.

## Conclusions

Over the past few years, AAV has risen to prominence as a viral vector system in human clinical trials, especially for the treatment of neurological and muscular diseases. Furthermore, AAV is also an ideal model for directed evolution studies because of its relatively simple genomic structure and the available viral engineering and production methods. However, there is still scope for further development and optimization of directed evolution strategies in modifying this vector. In particular, the practice of human gene therapy continuously presents new challenges for AAV and other viral vectors, such as addressing pre-existing immunity in human populations [49]. Driven by these needs, we expect further advancement in directed evolution of viral vectors, which will ultimately benefit the biomedical research and human health.

## Competing interests

The authors declare that they have no competing interests.

## Authors’ contributions

LY and XX participated in the writing of the manuscript and LY drew the figures. Both authors read and approved the final manuscript.
